# Human Support in App-Based Cognitive Behavioral Therapies for Emotional Disorders: Scoping Review

**DOI:** 10.2196/33307

**Published:** 2022-04-08

**Authors:** Emily E Bernstein, Hilary Weingarden, Emma C Wolfe, Margaret D Hall, Ivar Snorrason, Sabine Wilhelm

**Affiliations:** 1 Massachusetts General Hospital Boston, MA United States; 2 Department of Psychiatry Harvard Medical School Boston, MA United States

**Keywords:** digital health, mental health, cognitive behavioral therapy, coaching, guided, mobile app, emotional disorder, mobile phone

## Abstract

**Background:**

Smartphone app–based therapies offer clear promise for reducing the gap in available mental health care for people at risk for or people with mental illness. To this end, as smartphone ownership has become widespread, app-based therapies have become increasingly common. However, the research on app-based therapies is lagging behind. In particular, although experts suggest that human support may be critical for increasing engagement and effectiveness, we have little systematic knowledge about the role that human support plays in app-based therapy. It is critical to address these open questions to optimally design and scale these interventions.

**Objective:**

The purpose of this study is to provide a scoping review of the use of human support or coaching in app-based cognitive behavioral therapy for emotional disorders, identify critical knowledge gaps, and offer recommendations for future research. Cognitive behavioral therapy is the most well-researched treatment for a wide range of concerns and is understood to be particularly well suited to digital implementations, given its structured, skill-based approach.

**Methods:**

We conducted systematic searches of 3 databases (PubMed, PsycINFO, and Embase). Broadly, eligible articles described a cognitive behavioral intervention delivered via smartphone app whose primary target was an emotional disorder or problem and included some level of human involvement or support (*coaching*). All records were reviewed by 2 authors. Information regarding the qualifications and training of coaches, stated purpose and content of the coaching, method and frequency of communication with users, and relationship between coaching and outcomes was recorded.

**Results:**

Of the 2940 titles returned by the searches, 64 (2.18%) were eligible for inclusion. This review found significant heterogeneity across all of the dimensions of coaching considered as well as considerable missing information in the published articles. Moreover, few studies had qualitatively or quantitatively evaluated how the level of coaching impacts treatment engagement or outcomes. Although users tend to self-report that coaching improves their engagement and outcomes, there is limited and mixed supporting quantitative evidence at present.

**Conclusions:**

Digital mental health is a young but rapidly expanding field with great potential to improve the reach of evidence-based care. Researchers across the reviewed articles offered numerous approaches to encouraging and guiding users. However, with the relative infancy of these treatment approaches, this review found that the field has yet to develop standards or consensus for implementing coaching protocols, let alone those for measuring and reporting on the impact. We conclude that coaching remains a significant hole in the growing digital mental health literature and lay out recommendations for future data collection, reporting, experimentation, and analysis.

## Introduction

### Background

Smartphones are presently owned by 85% of the US population—a larger proportion than people who have access to computers or broadband subscriptions at home [1]. This high ownership rate represents substantial growth over the past 20 years, with rates in 2011 at just 35% [2]. Alongside this growth in smartphone ownership, there has been a corresponding proliferation in the recent development and deployment of app-based mental health treatments. In fact, in 2019, there were over 10,000 mental health apps available for download in the app market [3] with higher numbers likely available today.

There is good reason for the enthusiasm over app-based mental health treatments and skills-based approaches such as cognitive behavioral therapy (CBT) in particular that ostensibly lend themselves well to structured, standardized, self-paced platforms [4]. Smartphone-delivered therapies offer clear potential for addressing some of the most critical barriers to accessing mental health care, including prohibitive costs of treatment [5,6], patient-level logistical barriers (eg, need for time off work, transportation, and childcare) [7], and lack of access to providers who offer frontline evidence-based interventions such as CBT [8]. Indeed, in many parts of the United States there are fewer than 10 licensed psychologists per 100,000 people, with even fewer presumably offering evidence-based treatments [9]. In 2019—before the COVID-19 pandemic began—an estimated 1 in 5 adults were experiencing mental illness and even more subthreshold symptoms [10]. Prevalence rates are only increasing for younger cohorts and age groups [11,12]. This further underscores the enormous structural gap in available mental health care. Smartphones offer an opportunity to deliver impactful therapies that are readily accessible and widely scalable [13].

Although face-to-face CBT is the best studied psychosocial intervention for depression, anxiety, and related disorders, there is encouraging data showing that app-based CBT can be similarly effective [14]. Importantly, although app-based CBT offers substantial promise for addressing gaps in access to evidence-based mental health care, there are also key challenges compared with face-to-face treatment [13]. Most notably, app-based treatments often suffer from poor rates of sustained engagement, and efficacy and effectiveness studies are lagging behind app development. For example, within the IntelliCare suite of CBT skills apps, a study showed that the modal number of uses per app was once per user [15]. In an examination of engagement with the top 50 publicly available apps for depression and anxiety, more than half (63% for depression and 56% for anxiety) of the apps had no active monthly users [16]. Moreover, some engagement is likely a minimum requirement for an app to be effective. Regarding our understanding of efficacy and effectiveness, the large majority of mental health apps lack data altogether [17]. For example, in a review of available apps for anxiety and worry, 96.2% lacked efficacy data [18]. Thus, more systematic review and testing of app-based treatments is needed.

A frequent strategy advised and used by experts in digital mental health is to include human support [19,20]. However, how this has been implemented varies widely from light-touch reminders from lay support persons to in-depth, regular clinical attention and guidance from a specialized clinician. Note that in the literature, numerous titles are used to describe individuals who support patients in their use of app-based treatments, such as coach, therapist, specialist, and mentor. For clarity, the terms *coaches* and *guided* are used herein as umbrella terms to refer to human involvement in the delivery of app-based treatments. Coaches may enhance users’ accountability and motivation, potentially boosting engagement with otherwise impersonal app-based treatments. In support, a recent review of engagement in digital mental health interventions found that guided interventions had higher overall engagement compared with unguided interventions [21]. Coaches may also enhance the potency of app-based CBT, by delivering some of the treatment content, helping to personalize content for individual users, correcting how users implement skills, or answering questions. A meta-analysis of app-based mental health treatment efficacy showed that apps that offered coached guidance had larger effect sizes across several efficacy outcomes [4]. Altogether, both expert opinion [22] and initial, early evidence underscore the potentially critical role that coaches may play in enhancing the value of app-based CBT.

Despite the proposed benefits of incorporating coaches within smartphone CBT, we know surprisingly little at a systematic or empirical level about coaching. For instance, there are vastly different models of coach support being implemented across app-based CBT programs. We have little knowledge about whether professional-level support is necessary or if lay person or paraprofessional support may be equally beneficial (and more cost-effective and scalable). We do not know how much support (ie, dosing) is necessary or what type of support (eg, phone calls vs messaging and user- vs clinician-initiated communication) works best. In addition, we do not know how these recommendations would vary for different age groups (eg, adolescents and older adults). Ultimately, beyond a small number of initial studies, we know little about whether coaching reliably plays a role in enhancing engagement as intended or positively impacts the effectiveness of smartphone interventions. In fact, some studies have found that external supports are associated with worse treatment outcomes, as self-contained apps may be more comprehensive in their design or users may feel compelled to be more independently responsible for working through materials [23].

### Objectives

Each of these questions has direct implications for the scalability, effectiveness, and cost-effectiveness of app-based CBT. At a time when app-based CBT programs are being developed and deployed rapidly, we require a systematic, comprehensive evaluation of how coaching is currently implemented within interventions to guide the optimal design of future interventions and their scientific reporting. Given that the digital health field is increasingly moving toward app-based tools [24,25], this study centers specifically on app formats rather than collapse across internet and app-based approaches or focus on differences between them. To this end, the purpose of this study is to provide a scoping review of available data regarding the use of coaching in app-based CBT for emotional disorders, identify critical knowledge gaps, and offer recommendations for future directions [26].

## Methods

### Eligibility Criteria

Given the relative novelty of this topic, we opted to survey how coaching has been defined, implemented, and evaluated to date. Studies were included in this review if they met the inclusion criteria (Textbox 1).

**Inclusion criteria.**

**Inclusion criteria**

1. Describe or report on an intervention intended to improve an emotional disorder or concern as the primary aim. Targets include depression, anxiety, stress, psychological well-being, obsessive compulsive and related disorders, posttraumatic stress disorder or posttraumatic stress symptoms, and mood. Other targets necessitating meaningfully different interventions and thus potentially more different supports, such as serious mental illness (eg, substance use, bipolar disorder, and psychosis), a primary medical condition (eg, pain and sleep), and autism spectrum disorders, were excluded for this review.
2. Describe or report on an intervention based on cognitive behavioral therapy or skills, including cognitive (eg, restructuring and core beliefs) and behavioral approaches (eg, behavioral activation, exposure, and ritual prevention) Interventions based mostly or entirely on other approaches were excluded.
3. Describe or report on a treatment delivered entirely or in part via smartphone app outside of an in-person session. Interventions delivered exclusively in person or via the internet or that used smartphone apps only for scheduling, monitoring, reminders, or ecologic momentary assessment were excluded.
4. Describe or report on some aspect of human involvement or support (eg, coach or clinician) during app-based treatment.
5. Report was published in English and as a peer-reviewed journal article (eg, dissertations or conference abstracts were excluded). Published protocols were only included if a corresponding outcomes paper had not been published. Secondary analyses were included only if new analyses regarding human involvement were reported therein.
6. Report was published before April 1, 2021.

### Literature Search

**Figure 1:**
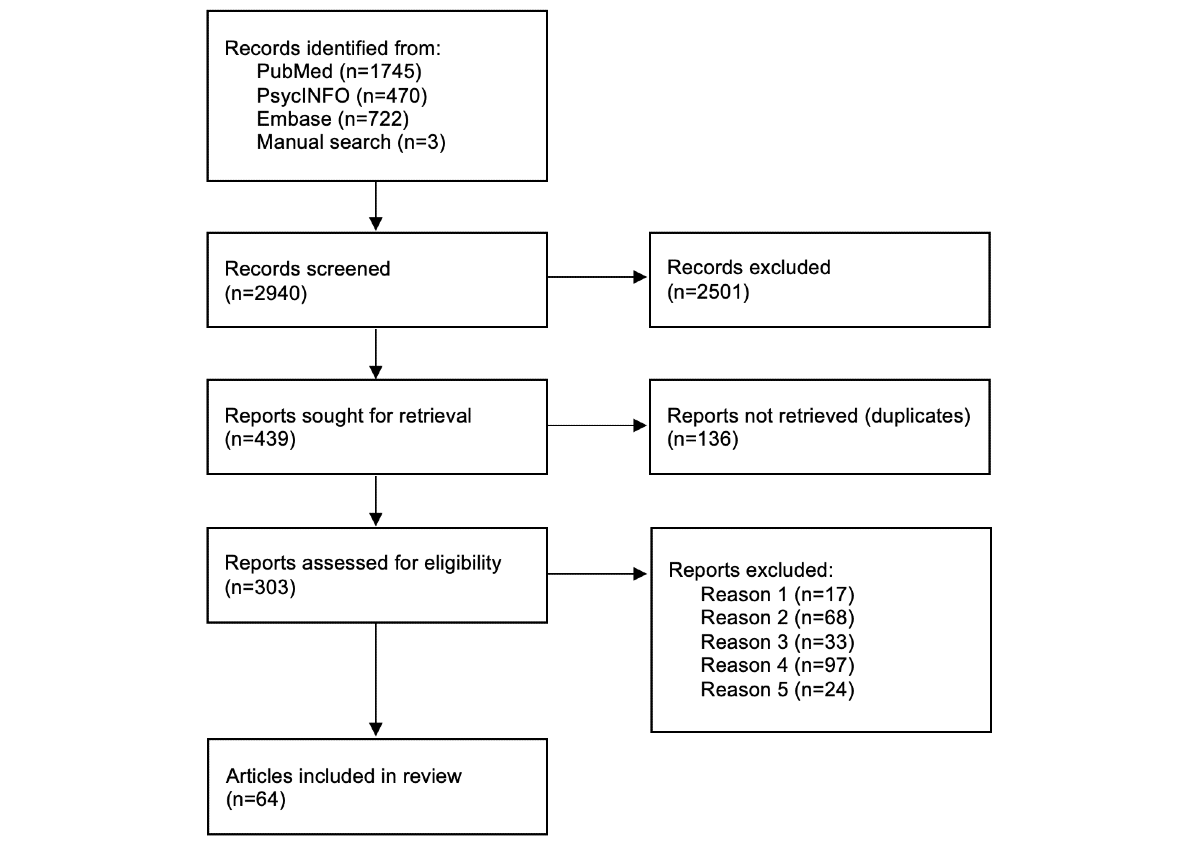
Flowchart of literature search. Reasons for exclusion: (1) primary aim or target is not an emotional disorder or concern, (2) intervention is not based on cognitive behavioral therapy or skills, (3) treatment is not delivered at least in part via smartphone app outside of an in-person session, (4) treatment does not include human involvement, and (5) published protocol or preliminary report is excluded given subsequent publication of outcomes paper.

To identify eligible articles, the authors conducted systematic searches in PubMed, PsycINFO, and Embase web-based databases using the following search terms in the title or abstract: *smartphone*, *mobile application*, *mobile app*, *app-based*, or *app-assisted*, in combination with *therapy*, *treatment*, *CBT*, or *iCBT*, and in combination with *depression*, *dysthymia*, *mood*, *MDD*, *anxiety*, *phobia*, *GAD*, *trauma*, *post-traumatic stress*, *posttraumatic stress*, *PTSD*, *obsessive compulsive disorder*, *obsessive-compulsive disorder*, *OCD*, *affective disorder*, *emotional disorder*, *emotional problem*, *stress*, *well-being*, or *wellness*. The database search and additional manual search (eg, searching reference sections of articles identified through database searches) occurred through April 1, 2021. In total, 2 researchers (EEB and ECW) read each title and abstract independently to screen for eligibility. In the event of disagreement, the article was included for the subsequent round of full-text review. In addition, two researchers (EEB and ECW) read the remaining articles in full, and exclusion required agreement. Uncertainties regarding inclusion at this level were discussed until consensus was reached. A flowchart summarizing this process is included (Figure 1). This report was informed by the PRISMA-ScR (Preferred Reporting Items for Systematic Reviews and Meta-Analyses extension for Scoping Reviews) checklist [27] (Table S1 in Multimedia Appendix 1).

### Data Review

Given the heterogeneity of resultant data and consistent with a scoping review, we provide a narrative rather than empirical synthesis of present evidence. Eligible articles were surveyed to determine how coaching is included in studies of app-based CBT. We examined the types of personnel serving as coaches (eg, bachelor’s level staff vs doctoral-level clinicians); the training, supervision, and standardization included with coaching; and the nature of the coaching itself. The latter includes the stated purpose or rationale for including a coach, the frequency and duration of coach contact, the method of contact (eg, phone call vs messaging), and the content of coaching (eg, encouragement vs teaching therapeutic content). Finally, we looked for evidence of whether coaching impacted users’ experience of treatment, engagement with the app, or clinical outcomes. In this effort, we also considered the consistency and absence of reporting in these domains.

To summarize some of these data, the following new categorical variables were created. *Coach qualifications* was operationalized as the minimum allowable degree or qualification for a coach; for example, trials including bachelor’s or master’s level coaches were labeled *bachelor’s level or above*. *Coach training* required of coaches was grouped as follows: (1) app- or study-specific training (ie, coaches underwent a seminar, workshop, or other formal training exercise to prepare them for the trial or coaches received detailed protocols or manuals to follow in their role), (2) app- or study-specific training plus ongoing supervision, (3) reliance on previous experience (ie, coaches are individuals with previous training, experience, or expertise in the therapy being delivered, such as behavioral activation or CBT for body dysmorphic disorder; no app- or study-specific training referenced), (4) previous experience plus ongoing supervision during the trial, or (5) ongoing supervision during the trial (ie, no other training or previous experience referenced). The *frequency* of coach communications was defined as daily, more than weekly (ie, 2-6 times per week), weekly, biweekly (ie, every other week), or less than biweekly (ie, less than twice per month). We summed across methods of communication; for example, if a coach made 1 phone call per week and sent 2 SMS text messages per week, the trial would be categorized as more than weekly frequency. *Method* of communication was defined as in-person (individual or group), phone (individual or group), or messaging (texting, chat, or email). We also sought to characterize *whether users were able to select the method*. If users had any autonomy in this regard (eg, users could select whether questions could be addressed via messaging or telephone), the trial would be labeled *yes*. We then labeled *who could initiate contact*: users only (eg, users reach out if questions arise or to confirm they have completed a module), coaches only (eg, communication only occurs during weekly planned phone calls), or both. We aim to characterize the *content* of coach communications with the following four categories: (1) encouragement (ie, reminders, motivational messages, technical support, or other attempts to increase sustained engagement and adherence), (2) encouragement plus questions (ie, coaches are available to respond with clinical advice when users reach out with questions about the treatment), (3) clinical intervention (ie, coaches initiate contact with all participants with the purpose guiding treatment, such as giving feedback on skills practice, recommending or prescribing specific skills or activities, and teaching or reviewing therapeutic concepts), or (4) a full course of treatment (ie, coaches administer in-person or telephone-based treatment as usual, such as behavioral activation, exposure with ritual prevention, or school counseling). Finally, *trigger for communication* was defined as whether certain conditions would necessarily prompt coaches to message participants (ie, score on a self-report measure, indication of suicidal ideation or self-harm risk, and not using the app for a set period).

In the event of missing data, we contacted corresponding authors to request clarification. Such inquiries were sent to 66% (42/64) of corresponding authors. We received responses from 55% (23/42). In the *Results* section, we report both the data included in the published articles and supplementary data provided by the authors who responded to our inquiries. However, patterns of missing data in the published literature are noted. Given these high rates of missing information, we examined patterns by publication year to explore whether reporting has changed over time.

## Results

### Overview

Our scoping review yielded 64 eligible articles (listed in Table 1). Of these 64 articles, 12 (19%) are published protocols without outcome data available as of the time of the review, 24 (38%) reported on randomized controlled trials, and 19 (30%) reported on open pilot or feasibility trials. A small number of articles described case studies (5/64, 8%), quasiexperimental designs (2/64, 3%), or field trials or real-world tests (2/64, 3%). Primary treatment targets included anxiety disorder or symptoms (12/64, 19%), depressive disorder or symptoms (24/64, 38%), transdiagnostic anxiety and depression symptoms (11/64, 17%), an obsessive compulsive or related disorder (5/64, 8%), posttraumatic stress disorder (PTSD) or posttraumatic stress symptoms (PTSS; 4/64, 6%), suicidality (1/64, 2%), and general mental health (eg, stress, well-being, and quality of life; 7/54, 11%). Treatment durations ranged from 3 to 24 (mean 8.83, SD 3.88; median and mode 8) weeks. Of the 64 studies, 54 (84%) were designed for adults, and 10 (16%) included children or adolescents.

A majority of articles reported on apps that were explicitly intended to be the primary mode of treatment delivery (52/64, 81%). An additional 13% (8/64) described apps that were designed to complement in-person treatment in also providing substantive content and skills implementation. The remaining projects varied based on provider preference and patient need. Notably, only 6% (4/64) of studies include experimentally varying the inclusion of a coach [53,69,79,89], one of which was a published protocol [89].

**Table 1:** Apps and their availability.

App (or suite of apps) name^a^	Trials, n^b^	Citation	Still active^c^	Commercially available^c^
¡Aptívate! (Behavioral Activation Tech, LLC)	1	Dahne et al, 2019 [28]	Yes	Yes
Agoraphobia Free (Health eLiving Partnership Ltd)	1	Christoforou et al, 2017 [29]	No	No
Anxiety Coach (Mayo Clinic)	2	Whiteside et al, 2014 [30]; Whiteside et al, 2019 [31]	No (on the web only)	No
Ascend (Meru Health)	3	Goldin et al, 2019 [32]; Economides et al, 2019 [33]; Economides et al, 2020 [34]	No	No
AWAKE	1	Berg et al, 2020 [35]	No	No
Behavioral Apptivation (Behavioral Activation Tech, LLC)	1	Dahne et al, 2018 [36]	Yes	No
BiP OCD (Stockholms läns landsting)	1	Lenhard et al, 2017 [37]	No	No
Boost Me (Voyage42)	1	Stiles-Shield et al, 2019 [38]	Yes	Yes
CBT Mobile-Work	1	Callan et al, 2021 [39]	No	No
CONEMO (The Latin America Treatment & Innovation Network in Mental Health)	1	Menezes et al, 2019 [40]	No	No
DCombat	1	Giosan et al, 2017 [41]	No	No
eQuoo (PsycApps Ltd)	1	Litvin et al, 2020 [42]	Yes	Yes
EVO (Akili Interactive Labs)	1	Arean et al, 2016 [43]	Yes	Yes
Get Happy Program (Developers of the Sadness Program)	1	Watts et al, 2013 [44]	Yes	No
GET.ON	2	Ebenfeld et al, 2020 [45]; Ebenfeld et al, 2021 [46]	No	No
Happy (independent programmers; specific developers not stated in paper)	1	Otero et al, 2020 [47]	Unclear	Unclear
HARUToday (Inha Intelligent Mobile Computing Lab)	1	Ham et al, 2019 [48]	Yes	Yes (as Haru: ASD)
Helpath (CICESE-UT3)	1	Martínez-Miranda et al, 2019 [49]	Yes	Yes (Google Play only)
iCanThrive (UVA Apps, LLC)	1	Chow et al, 2020 [50]	Yes	Yes (Google Play only)
IntelliCare (suite; Adaptive Health)	5	Chen et al, 2019 [51]; Graham et al, 2020 [52]; Mohr et al, 2019 [53]; Mohr et al, 2017 [54]; Orr et al, 2020 [55]	Yes	Yes
iPST	1	Arean et al, 2016 [43]	No	No
Journey to the West (The App Happy Project)	1	Lee et al, 2014 [56]	Yes	Yes
Kokoro (Flatt Steering Committee)	2	Mantani et al, 2017 [57]; Watanabe et al, 2015 [58]	No	No
Lantern (Thrive Network, Inc)	2	Newman et al, 2021 [59]; Oser et al, 2019 [60]	No	No
Meru Health Program (Meru Health)	1	Raevuori et al, 2021 [61]	Yes	Yes
MindClimb (Optio Publishing Inc)	1	Newton et al, 2020 [62]	Yes	Yes (Google Play only)
mindLAMP (Division of Digital Psychiatry)	1	Rauseo-Ricupero and Torous, 2021 [63]	Yes	Yes
Monsenso (Monsenso A/S CVR 35517391)	1	Tønning et al, 2021 [64]	Yes	Yes
nOCD (nOCD Inc)	1	Gershkovich et al, 2021 [65]	Yes	Yes
Pacifica (now Sanvello; Sanvello Health)	1	Broglia et al, 2019 [66]	Yes	Yes
Perspectives BDD (Koa Health)	1	Wilhelm et al, 2020 [13]	Yes	No
PsychAssist	1	Clough et al, 2015 [67]	No	No
PTSD Coach (US Department of Veterans Affairs)	3	Pacella-LaBarbara et al, 2020 [68]; Possemato et al, 2016 [69]; Tiet et al, 2019 [70]	Yes	Yes
RAW HAND	1	Hong et al, 2018 [71]	No	No
Run4Love (WeChat-based)	1	Guo et al, 2020 [72]	Yes	No
SmartCAT (University of Pittsburgh)	2	Silk et al, 2020 [73]; Pramana et al, 2014 [74]	Yes	Yes
SPARX (University of Auckland)	1	Werner-Seidler et al, 2020 [75]	Yes	Yes
Step-by-Step (World Health Organization)	1	Liem et al, 2020 [76]	Unclear	No
Stress Free (Thrive Therapeutic Software)	1	Christoforou et al, 2017 [29]	No	No
StressProffen (Oslo Universitetssykehus HF)	1	Børøsund et al, 2018 [77]	Yes	Yes
StudiCare Stress (Clinical Psychology and Psychotherapy Work Unit)	1	Harrer et al, 2018 [78]	No	No
Thought Challenger (part of the IntelliCare suite; Adaptive Health, Inc)	1	Stiles-Shields et al, 2019 [38]	Yes	Yes
VA apps (suite; US Department of Veterans Affairs)	1	Roy et al, 2017 [79]	Yes	Yes
Vida Health	1	Venkatesan et al, 2020 [80]	Yes	Yes
No name provided	10	Dagöö et al, 2014 [81]; Imamura et al, 2019 [82]; Ly et al, 2012 [83]; Ly et al, 2015 [84]; Ly et al, 2014 [85]; Springgate et al, 2018 [86]; Stolz et al, 2018 [87]; Uwatoko et al, 2018 [88]; Vázquez et al, 2018 [89]; Wilanksy et al, 2016 [90]	—^d^	—

^a^Developer included in parentheses where available.

^b^The numbers of trials do not add up to 64, as some articles reported on multiple apps.

^c^Data as of March 23, 2022.

^d^Not available.

### Who Is Providing Coaching?

Table 2 presents a summary of personnel details and training. Coach qualifications were not initially specified in more than one-third of the trials (24/64, 38%); however, authors of 50% (12/24) of these articles provided these data by email. Coaches ranged from upper-level undergraduate students, to bachelor’s level, graduate students, master’s level, and doctoral level. App- or study-specific training was initially described in only one-third of articles, most of which also included ongoing supervision for coaches. Note that details of these training materials were generally low; consequently, this group likely comprises wide variability in the time and resources devoted to coach preparation and ongoing quality control, including supervision or review of coaching transcripts or tapes. A number of studies using coaches with advanced degrees relied on relevant prior training or experience with the target population or in the target treatment. More than one-third of trials (23/64, 36%) did not describe whether there was any required training, supervision, or required previous experience for coaches; authors of 48% (11/23) of these articles provided data by email. Trials using bachelor’s level coaches consistently reported that coaches received explicit training and ongoing supervision. Graduate student coaches also frequently received this level of support, although it was not explicitly stated across all studies. About one-third of studies (23/64, 36%) described using coaching manuals, detailed protocols, scripts, or message or email templates to standardize at least some procedures, and authors of an additional 5% (3/64) of studies described using similar materials in email correspondence. This practice was not more or less common given the qualifications or study-specific training of coaches. Of the 64 studies, only 1 (2%) included formal fidelity checks for coaching [69].

**Table 2:** Coach characteristics (N=64).^a^

Characteristics		Value, n (%)
**Coach qualifications**		
	Undergraduate student or above	1 (2)
	Bachelor’s degree or above	17 (27)
	Graduate student or above	7 (11)
	Master’s level or above	17 (27)
	Doctoral level	10 (16)
	Not specified	12 (19)
**Coach training**		
	Prior professional experience required (ie, no study-specific training)	13 (20)
	Prior professional experience required with ongoing supervision (ie, no study-specific training)	2 (3)
	Study-specific training (ie, coaches received a manual or underwent a seminar, workshop, or other formal instruction)	18 (28)
	Study-specific training with ongoing supervision	16 (25)
	Supervision (ie, no study-specific training or prior experience specified)	2 (3)
	Not specified	13 (20)

^a^Counts reflect data included in the published articles and provided via email correspondence. Regarding coach qualifications, articles are grouped by the minimum training required for eligible coaches. For example, a trial using bachelor’s level coaches as well as first-year graduate students would be classified as bachelor’s degree or above.

### What Is the Stated Purpose of Coaching?

Although a number of studies provided no explicit rationale for including human support, the most common themes were for bolstering user motivation, engagement, and treatment adherence. Secondarily, availability for technical and clinical support or questions was often cited. When references were included, authors frequently drew on the broad internet-based CBT literature showing that technology-based interventions often fare better with some human support, including for user engagement or clinical outcomes [91-95]. When surveying articles for the content of coach communications, coaches were focused only on bolstering motivation and engagement through reminders and general encouragement in 23% (15/64) of studies. Coaches provided this encouragement and were available as needed for answering clinical questions in an additional 19% (12/64) of studies. It should be noted that encouragement comprises a wide range of approaches, including a simple reminder or motivational messages to which users were unable to respond as well as phone calls in which coaches worked to more actively engage and motivate participants. In 36% (23/64) of studies, coaches actively provided clinical intervention, including initiating review of therapeutic content with participants, giving feedback, or assigning specific activities or homework. Again, reports generally provided few details about the clinical content, such as the types of questions or strategies users requested, the extent of feedback or recommendations, or whether coaches would go beyond the content encompassed in the app (eg, offering non-CBT strategies). Coaches in 16% (10/64) of studies were tasked with delivering full courses of treatment to complement the app content (eg, weekly group therapy, individual CBT, or other therapeutic sessions). The role of coaches in 5% (3/64) of studies was unclear (eg, described coaches *providing support*), varied significantly by coach, or was not reported.

Few studies reported using triggers for coach communications, and most descriptions were vague (eg, *signs of deterioration* without further definition). A total of 5% (3/64) of studies noted that coaches would message participants had they not logged in to the app for a certain number of days. Of the 64 studies, 6 (9%) noted coaches monitoring or receiving alerts for indication of suicidality or self-harm risk (eg, Patient Health Questionnaire–9, item 9), and 5 (8%) noted that coaches would respond to signs of deterioration (eg, increase in score of depression scale). However, additional studies (33/64, 52%) did include at least some descriptions of information coaches had access to beyond their messages, calls, or sessions with participants to support or guide their communications. For example, coaches in 23% (15/64) of studies were able to view what participants had completed within the apps (eg, content viewed and activities logged); note that the level of detail available to coaches was frequently unclear (eg, the number of activities recorded vs what the specific activities were). Coaches in 3% (2/64) of the projects were able to see metrics of app use (eg, number of log-ins); of the 64 studies, 1 (2%) was unclear, describing users’ *progress*. In 13% (8/64) of studies, coaches could view results of self-report or other clinical measures. Finally, of the 64 projects, 3 (5%) allowed for access to both completed activities and clinical measures, and 3 (5%) allowed for access to both app use and clinical measures.

### How Do Coaches Communicate With Users?

Almost one-third (42/64, 66%) of included studies used messaging (ie, texting, app-based chat or messages, or email) as a method of communication between coaches and users, and 22% (14/64) of studies used messaging as the sole method. Phone or video calls were included in 50% (32/64) of studies and used as the sole method of communication in just 11% (7/64). In-person individual or group meetings were used in 25% (16/64) of studies and used as the sole method of communication in 11% (7/64). The most common approach was combining messaging with phone calls (22/64, 34%). Communication methods were unclear in 8% (5/64) of eligible studies. Of the 64 studies, only 9 (14%) allowed users to decide on the method of contact at least some of the time. Whereas many studies allowed patients to initiate contact (28/64, 44%), contact points were either preset or initiated only by the coach in just as many (29/64, 45%). Remaining projects did not provide sufficient or any information (7/64, 11%). A total of 11% (7/64) of projects reported on the number of messages sent by and to coaches, consistently finding that coaches typically initiate contact more than users [32,51,52,54,59,61,78].

**Figure 2:**
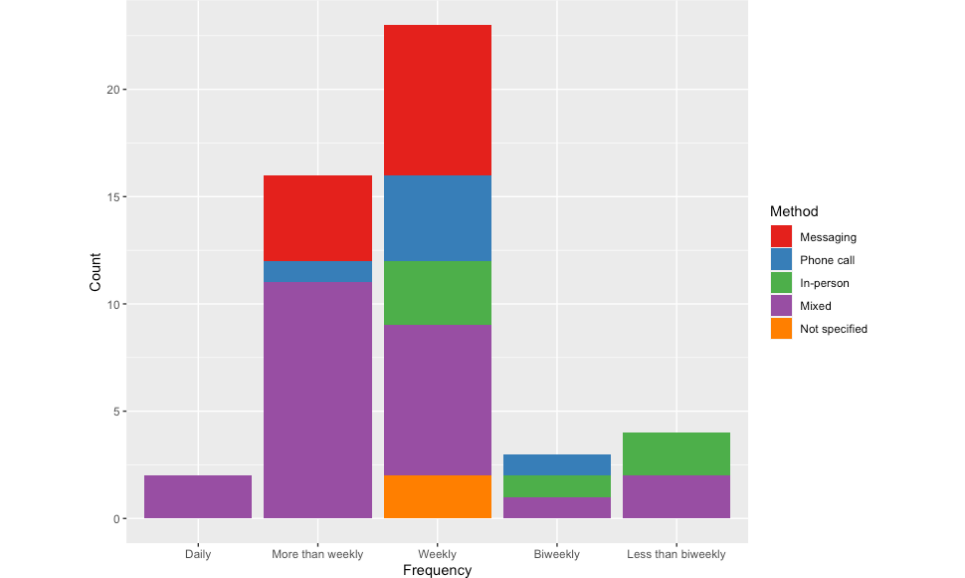
Coaching communication patterns. Counts reflect data included in the published studies and provided via email correspondence.

Frequency of communication varied widely from only twice across treatment to daily messages. Given the lack of data and clarity, we collapse across intended and actual reported frequencies. Notably, a large number of studies did not report on the intended or actual frequency (19/64, 30%); however, authors of 13% (8/64) of studies clarified their procedures via email. Coaches were most often in contact at least weekly (41/64, 64%), with more frequent communication largely driven by use of messaging as the sole or most used modality. The frequency of contact broken down by method of communication is visualized (Figure 2).

Discerning patterns related to coaches’ time was challenging. Given the lack of data and clarity, we collapsed across intended and actual durations. Among studies that offered at least some relevant data, weekly time per participant ranged from <10 to 60 minutes. In-person components tended to be equally distributed between standard session lengths (50-60 minutes) and 20- to 30-minute interactions. Phone calls were roughly evenly distributed among <10, <30, and 30 to 45 minutes. Longer phone calls were typically introductory contacts, and shorter calls tended to be for follow-up. Time devoted to messaging was rarely quantified; the few estimates (6/64, 9%) ranged from an average of 2.2 minutes per participant per week to upward of 30 minutes per participant per week. Unsurprisingly, coaches providing some level of clinical intervention typically spent more time per participant than coaches providing only encouragement and reminders. Notably, these patterns should not be overinterpreted, as more than half of the studies did not include any information regarding the amount of time coaches spent in total or per contact (37/64, 58%; in total, 10 authors were able to provide at least partial, additional estimates when contacted) and many more did not break down time commitments by method of communication. In addition, 27% (17/64) of studies offered no information regarding frequency or duration of coach communications; however, 11% (7/64) of studies were able to provide more information regarding one or both in follow-up communications.

### What Is the Impact of Coaching?

#### Overview

Ultimately, only 22% (14/64) of studies considered whether the presence of a coach or the level of coaching received contributed to their intervention (Table 3). In addition, of the 64 studies, 6 (9%) included qualitative feedback from users [32,35,40,46,70,84], 5 (8%) examined the impact of coaching on engagement [38,51,53,79,80], and 7 (11%) analyzed the relationship between coaching and outcomes [35,52,53,59,69,79,80].

**Table 3:** Summary of studies analyzing the role of coaching in treatment engagement and outcomes.

Citation	Analysis	Finding	Sample	Treatment	App	Coach^a^	Communication^b^
Ebenfeld et al, 2021 [46]	Qualitative	Insufficient coaching as reason for dropout	92 adults, diagnosed panic disorder, majority women, White, mean age 38 (SD 10.4) years	CBT^c^ for panic	GET.ON	Bachelor’s level	Weekly messages
Goldin et al, 2019 [32]	Qualitative	Positive regard for coaching	2 studies, 22 and 95 adults, at least mild depressive symptoms, majority women, White, mean age 23.2 (SD 1.1) and 32 (SD 9.9) years	MBSR^d^ and MCBT^e^ exercises for depression	Ascend	Master’s level	Messaging or phone calls 2-3 times per week
Ly et al, 2015 [84]	Qualitative	Positive regard for coaching	12 adult, diagnosed with MDD^f^, 50% (6/12) women, mean age 38 (SD 14) years	BA^g^ for depression	Not reported	Graduate students	At least weekly messaging
Menezes et al, 2019 [40]	Qualitative	Positive regard for coaching	66 adults, at least moderate depressive symptoms and comorbid hypertension or diabetes, majority women, aged 41-60 years	BA for depression	CONEMO	Nurse or nurse assistant	Weekly in-person meetings or phone calls
Tiet et al, 2019 [70]	Qualitative	Positive regard for coaching	29 adults, probable PTSD^h^ diagnosis, majority men, White, median age 61 years	CBT skills for PTSD symptoms	PTSD Coach	Paraprofessional	Phone calls every other week
Berg et al, 2020 [35]	Qualitative and outcomes	Positive regard for coaching; mixed effects for outcomes	38 adults, cancer survivors, majority women, White, mean age 32 (SD 5.5) years	CBT for general mental health	AWAKE	Master’s level	Weekly phone calls; twice-weekly texts
Mohr et al, 2019 [53]	Engagement and outcomes	Mixed effects for engagement; mixed effects for outcomes	301 adults, at least moderate depressive symptoms or mild to moderate general anxiety symptoms, majority women, White, mean age 37 (SD 12) years	CBT skills for transdiagnostic depression or anxiety	IntelliCare (suite)	Bachelor’s level	Initial call; optional midtreatment call; 2-3 messages per week
Roy et al, 2017 [79]	Engagement and outcomes	Positive effects for engagement; slower symptom change	144 adults, subthreshold PTSD symptoms, majority men, White, mean age 33 (SD 11) years	CBT skills for PTSD symptoms	VA apps (suite)	Doctoral level	Introductory meeting; daily messages
Venkatesan et al, 2020 [80]	Engagement and outcomes	Positive effects for engagement; mixed effects for outcomes	323 adults, mild to moderate depressive or general anxiety symptoms, majority women, mean age 36 (SD 9) years	CBT skills for transdiagnostic depression or anxiety	Vida Health	Master’s level	Weekly phone calls; messaging as needed
Chen et al, 2019 [51]	Engagement	No effects for engagement	98 adults	CBT skills for transdiagnostic depression and anxiety	IntelliCare (suite)	Bachelor’s level	Initial call; 2 messages per week
Stiles-Shields et al, 2019 [38]	Engagement	No effects for engagement	30 adults, at least moderate depressive symptoms	BA or cognitive restructuring for depression	Boost Me; Thought Challenger	Master’s level	Weekly phone calls or emails
Graham et al, 2020 [52]	Outcomes	No effects for outcomes	146 adults, at least moderate depressive or mild to moderate GAD^i^ symptoms, majority women, White, mean age 42 (SD 13.8) years	CBT skills for transdiagnostic depression or anxiety	IntelliCare (suite)	Bachelor’s level	Initial call; optional midtreatment call; 2 messages per week
Newman et al, 2021 [59]	Outcomes	No effects for outcomes	100 college students, self-reported GAD, majority women, White, mean age 21 years	CBT for anxiety	Lantern	Bachelor’s level	Phone calls or messaging as needed
Possemato et al, 2016 [69]	Outcomes	Positive effects for outcomes	20 veterans, likely PTSD diagnosis, majority men, mean age 42 (SD 12) years	CBT skills for PTSD symptoms	PTSD Coach	Master’s level	In-person meetings or phone calls every other week

^a^Coach: minimum required degree or qualification to be in the supportive human role.

^b^Frequency and method of coach contact.

^c^CBT: cognitive behavioral therapy.

^d^MBSR: mindfulness-based stress reduction.

^e^MCBT: mindfulness-based cognitive behavioral therapy.

^f^MDD: major depressive disorder.

^g^BA: behavioral activation.

^h^PTSD: posttraumatic stress disorder.

^i^GAD: generalized anxiety disorder.

#### Qualitative Feedback

Users largely shared positive impressions about coaching. Berg et al [35] reported on master’s level coaches who engaged with users via weekly phone calls and twice-weekly SMS text messages over the course of 8 weeks. Treatment focused on promoting hope, positive mood, and behavioral goals among young adult cancer survivors. Note that emotional well-being, rather than managing physical health or related processes, was the primary treatment target. Coaches aimed to review content from the app, practice skills with users, assign homework, and offer encouragement. After the treatment, 94% of users recommended that coaching remain part of the program moving forward. Similarly, 90% of users in the study by Menezes et al [40] reported coach support to be an important treatment component. In this study, coaches were nurses or nursing assistants offering weekly phone or in-person meetings over 6 weeks to answer questions and offer encouragement for individuals with depression and comorbid hypertension or diabetes. Goldin et al [32] asked users (adults with depressive symptoms) to rate the value of coaching at weeks 1, 3, and 6 and follow-up time points. Coaches were master’s level or above professionals who engaged with users via messaging or phone calls 2 to 3 times per week over 8 weeks. Coaches aimed to check in, answer questions, and facilitate a group chat among users. Users rated the value of coach interactions on average 4.13 out of 5. Tiet et al [70] used paraprofessionals (graduate students) as coaches, who offered users with likely PTSD six 5-10–minute phone calls for technical support and encouragement over 12 weeks. They found that 91% of users reported that coach support was at least somewhat helpful and 74% reported that coach’s support positively impacted the frequency and consistency of their app use. In a follow-up, qualitative analysis of the primary report by Ly et al [85], researchers asked for feedback from patients with depression regarding how they perceived having up to 20 minutes of weekly messaging with a coach related to their treatment engagement and outcomes. Coaches in this study offered encouragement, general education, and weekly feedback on patients’ written reflections [85]. Users shared that coaching was crucial to their app use and treatment effects, and most indicated that more frequent and more personalized contact would be preferable [84]. Both Ly et al [84] and Ebenfeld et al [46] described 1 participant each who withdrew from the trial citing insufficient direct contact with their coach. In the latter study, coaches were at least bachelor’s level staff members and offered patients with panic disorder weekly feedback messages over 6 weeks [46].

#### Engagement

In total, 3 trials of the IntelliCare suite or its individual apps, designed to help patients with depressive or anxious symptoms, described the relationship between coaching and objective metrics of engagement [38,51,53]. Chen et al [51] examined how responsive patients were to coaches’ messages. In their trial, coaches offered an initial call (30-45 minutes) and sent at least two messages per patient per week over 8 weeks to answer questions, provide recommendations and encouragement, and help patients problem solve. Responsiveness (ie, number of messages patients responded to) was ultimately unrelated to engagement with the apps, operationalized as the number of times apps were opened. In the study by Stiles-Shields et al [38], master’s level coaches offered weekly encouraging phone calls or emails (<10 minutes per patient per week) over 6 weeks. Similarly, they found that neither the number nor duration of these contacts correlated with app use, including number of app launches and number of activities logged. Mohr et al [53] manipulated whether patients had access to a coach at all. Over 8 weeks, coaches offered encouragement and answered patient questions via an initial call (30-45 minutes), optional midtreatment call (10 minutes), and 2 to 3 messages per patient per week. Patients in this coached condition did download more skills apps but did not engage in more consistent app sessions than patients who were not given coaches. When testing the VA suite of apps for patients with PTSS, Roy et al [79] found that patients who were given access to guidance from a doctoral-level coach (eg, directing users to particular skills) did self-report using the apps more frequently compared with the group that received nondirective contact, particularly for apps providing psychoeducation and tools for controlled breathing. Over 6 weeks, coaches conducted introductory in-person meetings to go over CBT skills followed by daily messages to guide app use. Finally, Venkatesan et al [80] tested the Vida Health app among adults with mild to moderate depression or anxiety. Master’s level therapists provided weekly 30-minute consultations via video or phone call over 12 weeks and were available for additional in-app messaging as needed. The number of lessons or activities users completed in the app was strongly correlated with the number of consultations they completed and moderately correlated with the number of messages they sent to their therapists. Overall, across studies, there was no consistent pattern regarding the effect of coaching on app engagement. To build our understanding of the value of coaching on engagement, more studies that adopt a randomized (ie, varying presence or quantity of coaching) design are necessary. In addition, there was no consistent operationalization of engagement either, further challenging our ability to draw conclusions.

#### Outcomes

Outcomes data were similarly limited and mixed. A total of 3 papers reported some positive effects of coaching, 2 (67%) of which compared groups of users with and without access to a coach. First, Possemato et al [69] tested the PTSD Coach app for veterans that screened positive for a likely PTSD diagnosis. Participants in the coaching condition received biweekly in-person or phone sessions over 8 weeks in which at least master’s level clinicians introduced and reviewed content and assigned homework. Compared with participants in the self-guided condition, participants with coaches demonstrated larger gains for PTSD symptoms, depression, and quality of life. In the aforementioned study of the IntelliCare suite of apps by Mohr et al [53], users with access to a coach exhibited larger declines in anxiety (General Anxiety Disorder scale–7 scores) than peers without coaches but exhibited no differences for depression (Patient Health Questionnaire–9 scores), the other primary outcome. Similarly, in the aforementioned study by Berg et al [35], the number of phone calls users completed with a coach was associated with greater reductions in days of alcohol use and improvements in pain-related functioning, but there were no differences for other primary outcomes including hope, depressive symptoms, or other domains of quality of life. In addition, in the aforementioned study by Venkatesan et al [80], therapist consultations but not messages predicted decline in depressive symptoms, and there were no reported effects for anxiety.

In contrast, 2 papers reported no effects for level of coaching; notably, however, all participants did receive a coach in these studies. Newman et al [59] offered 12 weeks of app-based CBT for anxiety to college students. Coaches were at least bachelor’s level study staff and provided as-needed phone calls and messaging to support goal setting, provide encouragement and feedback, and answer questions throughout treatment. The number of messages between coaches and users was ultimately not associated with symptom change. Graham et al [52] also reported that the number of messages exchanged with a coach was not associated with symptom change. In their study, at least bachelor’s level coaches provided initial phone calls, optional midtreatment phone calls, and ≥2 messages per week to users experiencing depressive or anxious symptoms over 8 weeks. Coaching focused on goal setting, making recommendations for skills to practice, and offering encouragement. Finally, the aforementioned trial by Roy et al [79] of the VA apps for PTSS found that access to a coach offering treatment guidance actually correlated with slower symptom change. Authors speculated that this effect could be due to the control condition (nondirective messages comprising nonspecific positive aphorisms) possibly being perceived as encouraging as well or the fact that the 2 groups engaged similarly with the more active skills apps (eg, social engagement and relaxation exercises). Similar to the aforementioned findings, reports yielded mixed results for coaching’s effect on outcomes, and more research that is specifically designed to answer questions about the effect of coaching on outcomes is needed to clarify mixed findings.

It is notable that in research studies, participants may have contact with study staff outside of these intended supports (eg, communicating with research assistants about scheduling assessments). Such contact could be perceived as encouraging or could increase a participant’s sense of accountability, thus potentially accounting for differences in observed engagement and clinical outcomes. Consequently, we also reviewed these studies for additional touch points. Studies did not report on this in depth and largely described pretreatment contact, if any. Studies that had study staff systematically reach out to participants—including for a pretreatment [35,38,69,79] or posttreatment [59] phone call or interview or to administer periodic web-based assessments during treatment [52]—did not have consistently better engagement or outcomes than those that did not report such additional contact [51,53,80].

### Has Reporting Improved Over Time?

We present the proportion of papers with missing data or descriptions of various aspects of coaching by year (Table 4). Overall, time spent communicating with users (*duration*) was the most often omitted data point, followed by coach training, coach qualifications, and frequency of communications. It is difficult to determine statistically meaningful trends owing to the increasing number of publications in recent years; however, it does not appear that reporting has substantially changed over time.

**Table 4:** Proportions of missing data over time.^a^

Dimension	2012 (n=1), n (%)	2013 (n=1), n (%)	2014 (n=5), n (%)	2015 (n=3), n (%)	2016 (n=3), n (%)	2017 (n=6), n (%)	2018 (n=7), n (%)	2019 (n=16), n (%)	2020 (n=17), n (%)	2021 (n=5), n (%)	Total (N=64), n (%)
Qualifications	1 (100)	1 (100)	2 (40)	0 (0)	1 (33)	3 (50)	3 (43)	4 (25)	5 (29)	0 (0)	20 (31)
Type of training	1 (100)	1 (100)	2 (40)	1 (33)	1 (33)	4 (67)	2 (29)	4 (25)	4 (24)	1 (20)	21 (33)
Frequency	1 (100)	1 (100)	2 (40)	0 (0)	1 (33)	1 (17)	2 (29)	3 (19)	7 (41)	1 (20)	19 (30)
Duration	1 (100)	0 (0)	3 (60)	2 (67)	2 (67)	1 (17)	5 (71)	10 (63)	12 (71)	4 (80)	40 (63)
Method	1 (100)	0 (0)	2 (40)	0 (0)	0 (0)	0 (0)	0 (0)	1 (6)	2 (12)	0 (0)	6 (9)
Selection	1 (100)	0 (0)	2 (40)	0 (0)	0 (0)	0 (0)	0 (0)	1 (6)	1 (6)	0 (0)	5 (8)
Initiation	1 (100)	0 (0)	1 (20)	1 (33)	0 (0)	0 (0)	1 (14)	1 (6)	2 (12)	0 (0)	7 (11)
Content	1 (100)	0 (0)	1 (20)	0 (0)	0 (0)	0 (0)	1 (14)	1 (6)	2 (12)	2 (40)	8 (13)

^a^Values reflect the proportion of studies in a given year that did not report a given facet of their coaching protocol or results. For example, 40% (2/5) of the published studies from 2014 did not describe the qualifications of their coaches in the text.

### What Is the Current Status of Included Apps?

Table 1 includes a summary of all apps reported on in the eligible studies as well as their current status. As of the time of review, only 26 of the 44 (59%) named apps were active (22/26, 85% available commercially).

## Discussion

### Principal Findings

The primary aim of this scoping review is to characterize patterns of coaching in guided app-based CBT for emotional disorders or concerns and to identify knowledge gaps. Digital mental health is a young but rapidly expanding field with enormous potential to improve the reach of evidence-based care. Researchers across the reviewed articles offered numerous approaches to encouraging and guiding users involved in these treatments (eg, prescheduled weekly phone calls to review content, daily encouraging messages, and communication as needed). Such efforts are foundational as researchers continue to improve the flexibility and accessibility of psychotherapy. However, with the relative infancy of app-based treatment approaches, this review found the field has yet to develop standards or consensus for measuring and reporting on coaching. For example, nearly half of the trials did not specify the level of training the coaches received, including their degrees, instruction, or experience with the treatment or the target population. A large number of studies also did not define the role of coaches in the intervention itself. Only a minority of studies reported on the actual number of phone calls, sessions, or messages users attended or received and even fewer the actual time commitments of coaches. In addition, little information was provided on the nature of supervision or ensuring that coaches adhered to their specified roles. Most immediately, these missing data limit our ability to identify meaningful trends in coaching for app-based treatments. From this review, it appears that no one type of coach, level of training, stated purpose of the coaching, method of coaching, frequency of communication, or duration of communication is currently the norm. This heterogeneity holds true when examining subsets of treatments, such as apps designed for specific populations (eg, children vs adults, and depression vs general mental health) or specific treatment lengths.

More consistent reporting moving forward will allow for replication of interventions across sites, a prerequisite for establishing treatment effectiveness, determining under what conditions and in what forms coaching adds clinical utility and thus amassing an evidence base for best practices. In this vein, few studies have evaluated the effects of coaching in their analyses. Qualitative data did suggest that users largely appreciate having access to a coach, describing this human element as helpful and even critical to their experience. This aligns with a number of recent reviews and meta-analyses finding that guided app-based treatments—or those with some human support—generally have better completion rates and treatment outcomes than self-guided ones [4,96,97]. However, when we then examined articles that present direct comparisons of guided versus unguided versions of the *same* app-based treatment or considered *level* of coaching within a trial, the quantitative data offered a murkier story. Although a number of studies found positive results, 1 project reported negative effects that guided treatment led to slower symptom change than unguided treatment, and half of studies reported no effects at all.

Looking again to the internet-based CBT literature, this inconsistency aligns with other reviews that have found that supported and unsupported digital interventions may not systematically differ [98,99] and that more coach time may not linearly lead to better outcomes [100]. Nevertheless, the prevailing wisdom is that the provision of human support can improve user engagement and outcomes in digital mental health [91-95]. This sentiment has been echoed in the development of app-based approaches. However, closer inspection shows that these highly cited reviews of guided versus unguided digital interventions similarly suffer from a lack of detail. *Guidance* is broadly and often unclearly defined [91], and decisions are often based on intuition or feasibility rather than evidence-based guidance or coaching protocols [101]. Heterogeneity in these reviews’ conclusions about the importance of coaching could be accounted for by the heterogeneity in how authors choose to define *guidance*, CBT, and the populations studied [102].

Taken together, we cannot draw strong conclusions from this study’s data, not only because of the small number of studies available but also as there are too many differences across them, including the target population, symptom severity, precise type and duration of treatment, method and frequency of coach communication, and type of coaches and coaching offered. How authors chose to quantify coaching, engagement, and outcomes was similarly variable. We conclude that coaching remains a significant hole in the rapidly growing digital mental health literature.

### Future Directions

#### Increase Data Collection and Reporting

Thus, the first takeaway from this review is that more consistent, thorough, and standardized reporting is needed. Recommended dimensions of coaching are included as a checklist for future use in Table S2 in Multimedia Appendix 1. At minimum, the following data should be included in guided digital mental health trials moving forward (highlighted in Textbox 2): (1) Coach qualifications (if any). App-based treatments that are successfully guided by bachelor’s level or paraprofessional coaches will be less expensive and more scalable than app-based treatments requiring guidance by doctoral-level coaches, underscoring the importance of these data. (2) Details regarding the materials, training, or other support provided to coaches. (3) Instructions provided to coaches regarding the purpose and boundaries of their roles and expectations set for participants in this regard. (4) The modalities and timing of coach communications—planned and actual. A coach’s time is likely to be one of the most expensive parts of scaling an app-based treatment; consequently, studies should more consistently set expectations around coaches’ time and measure actual time spent performing this role. (5) Adherence metric. Although feasibility may limit the thoroughness of adherence measures (eg, availability of independent raters to evaluate tapes or text), some effort should be made to assess whether coaching largely followed the intended parameters.

**Highlighted recommendations: key recommended dimensions to report regarding human support in app-based therapy.**

**Recommendations**

1. Coach qualifications: what criteria were used to select eligible coaches?
2. Coach training: what written, live, or other training opportunities did coaches receive? Were coaches supervised in their work?
3. Coach instructions: what were coaches instructed to do in their role, including the purpose, nature, or boundaries of their communications with users?
4. Logistics: how, how often, and for how long were coaches in communication with users?
5. Adherence: to what extent did coaches' actual communications with users match the expected content, modalities, frequencies, and durations?

Beyond the aforementioned *must haves*, additional data, when feasible, would also be helpful: (1) differentiating communications initiated by users versus coaches; (2) user preferences for modality, frequency, timing, and content of coach communications; (3) whether the coaching that users received matched these preferences; and (4) audio recordings or transcripts of coach communications for adherence or quality ratings as well as more detailed future analysis. Notably, data from internet-based therapies reveal that coaches often deviate from guidelines, particularly for more complex interventions (eg, offering feedback, facilitating understanding, and reinforcing practice), with less engaged users, and when coaches have less specialized experience [103]. Such norms in reporting would allow for more accurate evaluation of treatments, identification of common challenges, greater comparison across trials, and dissemination into real-world settings, including cost-benefit analysis. With more data, guidelines for assessing the quality of coaching protocols would also be possible [104].

#### Experimentally Evaluate the Effects of Coaching

The second takeaway is that more explicit testing of coaching effects is needed. This includes additional randomized controlled trials comparing guided and unguided versions of the *same* treatment, as well as experimentally varying the type or amount of coaching users receive and exploring moderators of coaching effects. This should be done in a hypothesis-driven way as researchers consider the target population (eg, age, diagnosis, and illness severity) and the content of the app itself (eg, level of detail, personalization, and structure). Microrandomized trials could be particularly powerful for these types of questions, allowing investigators to systematically test multiple small but potentially impactful and expensive types of communication at various decision points [105]. Real-world testing will be a critical contribution to this literature as well. Human support is most frequently conceptualized as a tool for boosting adherence; individuals enrolled in clinical trials of unguided app-based treatments more often than not still receive at least some contact with study staff, which could be serving an unanticipated but similar purpose to reminders and encouragement from coaches [102]. In fact, in some of the reviewed articles, it was challenging to differentiate such clinical trial implementation processes from intended reminders or supports that were of extremely light touch [86]. Without these added contacts, outcomes could look quite different. Indeed, outside of controlled research settings, engagement data are less encouraging; for example, an analysis of the most popular apps addressing anxiety, depression, or emotional well-being—defined as the 93 apps with at least 10,000 downloads—revealed a median daily engagement rate of just 4% [106]. Interestingly, the few studies included in this review that examined the relationship between coaching and engagement or outcomes did not report significant study staff contact outside of care delivery (largely before treatment). However, scientific papers rarely describe this type of extra study contact, let alone in detail. Better tracking all forms of intended and unintended support in the future would help address this question.

It is likely that some dimensions of coaching needs will also vary by individual user characteristics or preferences as well as other dynamic variables, such as time in treatment, status of treatment response, or how a user is using coaching (eg, reassurance seeking, problem-solving barriers to skills use, and accountability checks). For example, coaching may make the most difference for users with more severe symptoms [94] or early on in treatment [44], or the frequency or tone of communications may need to change over time to remain engaging [107]. Conversely, some dimensions may matter less. For example, research on internet-based interventions have shown that the qualifications of coaches are weak predictors of outcomes [101,108]; this review also did not identify any patterns in results based on coach qualifications.

In addition, in this review, many studies comprised small or homogeneous samples of largely White, female, and relatively young adult participants. Results could therefore be meaningfully different with larger, more diverse populations. First, age may be an important factor, although the included studies did not test this moderator. For example, quantity of coaching could be more important to older adults than to their younger counterparts. Studies have found that desire for human contact and technology literacy barriers can be deterrents for mental health app use, particularly among older adults; thus, more coaching may be beneficial for these users [55,109]. Moreover, others have found older adults to be more engaged with digital coaches than younger ones [110]. In contrast, rather than necessarily wanting more contact, adolescents may prefer different approaches; to illustrate, integrating peer support and strength-based messaging may be preferable [111]. Younger users may also be more open to virtual avatars providing guidance and support or mirroring their experience [112]. In general, more work is needed around age-appropriate messaging, for both coaches and in-app content [113,114]. Second, there is some evidence that digital interventions may reduce longstanding disparities in treatment access, response, and dropout between White and racial and ethnic minority patients [115]. Understanding how coaching could strengthen this trend as well as reach other underrepresented potential users, including sexual and gender minority individuals or older adults, should be a priority. For example, researchers may closely examine patterns of user preferences, use coaches to increase the credibility of technology-based options, support technological literacy, or actively work with individuals to adapt skills to their unique stressors or contexts [116].

#### Test Hypothesized Targets

Relatedly and third, consistent with broader efforts in psychotherapy research, more mechanism- or process-oriented evaluation is needed [117]. There should be testable hypotheses regarding a coach’s function in app-based treatment, such as enhancing motivation, reducing a specific barrier, increasing comprehension, or supporting use of skills. In this way, the number of minutes or messages with a coach alone may not be the best metric of coaching quality, nor should end-of-study symptom severity or diagnoses be the sole outcome variables. For example, the supportive accountability model [19] and efficiency model of support [118] offer compelling frameworks for designing coaching protocols for digital interventions. Supportive accountability predicts that treatment adherence will increase if coaches are viewed as trustworthy, knowledgeable, helpful, and collaborative; in addition, coaches should increase salience and perceived utility or personal relevance of new behaviors for individual users to increase their motivation and thus engagement [19] The efficiency model argues that human support should directly address specific failure points of digital treatments (usability, engagement, fit, knowledge, and implementation) [118]. Multiple studies in this review reported implementing protocols based on these principles [38,51,53,54,78]. However, to the best of our knowledge, none tested the impact beyond the overall treatment effects. Fortunately, clear, testable targets are outlined in both models. These can be operationalized and researchers should test, for example, whether and how coaches effectively mitigate these failure points (and, perhaps, do so better than less time- and resource-intensive solutions), and whether doing so directly leads to better treatment outcomes as intended.

In this effort, researchers can capitalize on digital technologies to evaluate these variables and their interactions, including ecological momentary assessment, text analysis, machine learning, or other dynamic, idiographic approaches [119]. Leveraging these insights could lead to not only more targeted, streamlined coaching but also more effective automated support, a less expensive, more scalable alternative. Currently, although coaching ostensibly outperforms automated messages or reminders in enhancing engagement with digital therapies, the latter may still provide clinically meaningful benefits and are appreciated by users [21] and fully automated chatbots and other conversational agents are becoming more sophisticated [120]. Process-focused and individualized data of these kinds could support better algorithms dictating the timing and content of automated coaching. It remains possible that automated coaching that is based on high-quality data-driven learning and that is by nature available continuously could become as or even more effective than live coaches.

### Limitations

Additional limitations of this scoping review merit noting. Because of the heterogeneity among trials and extent of missing data, a more systematic review including evaluation of the quality of studies or meta-analysis of results was not possible. The aim of this scoping review is to assess current practices and encourage new norms in reporting of guided app-based treatments to allow for this much needed next step. Relatedly, although search terms and eligibility criteria were intentionally broad and manual searches were conducted, it is also possible that some projects were missed. Present data were numerically skewed toward depression-focused treatments, a pattern consistent with other scoping and systematic reviews of mobile and app-based mental health treatments in general. It is possible that with more work on other patient populations, different or clearer trends could emerge.

### Conclusions

The promise of digital treatments is scalability. Whether an app requires a bachelor’s level versus doctoral-level coach or a coach at all, 2 versus 45 minutes of a coach’s time per patient per week or per month, extensive training and supervision, or messaging versus face-to-face conversation to be engaging or effective would all substantially impact how scalable it can be. After reviewing the literature on guided app-based CBT, it is clear that guided interventions can be operationalized with startling diversity. Consequently, it is time to retire the notion that coaching of any kind will improve outcomes. Not only do data not consistently support this conclusion, but also the conclusion itself does not offer sufficient direction for thoughtful design and implementation of new treatments. What does seem clear is that users typically respond positively to the availability of a coach. How much ensuing interactions actually change their behavior or outcomes remains an open question. Fortunately, apps open up previously unfeasible, diverse, and creative ways to reach patients. In parallel, new technologies open up new possibilities for optimizing their impact. To take advantage of these opportunities, as a field, we must increase transparency around coaching and prioritize this treatment component as a focus in future research.

## Appendix

Multimedia Appendix 1 PRISMA-ScR (Preferred Reporting Items for Systematic Reviews and Meta-Analyses extension for Scoping Reviews) checklist and checklist for recommended reporting of human support in digital mental health treatment.

## Abbreviations

CBT: cognitive behavioral therapy
PRISMA-ScR: Preferred Reporting Items for Systematic Reviews and Meta-Analyses extension for Scoping Reviews
PTSD: posttraumatic stress disorder
PTSS: posttraumatic stress symptoms
